# Designing Spin Symmetry for Altermagnetism with Strong Magnetoelectric Coupling

**DOI:** 10.1002/advs.202503235

**Published:** 2025-06-17

**Authors:** Wei Sun, Wenxuan Wang, Changhong Yang, Shifeng Huang, Ning Ding, Shuai Dong, Zhenxiang Cheng

**Affiliations:** ^1^ Shandong Provincial Key Laboratory of Green and Intelligent Building Materials University of Jinan Jinan 250022 China; ^2^ School of Material Science and Engineering University of Jinan Jinan Shandong 250022 China; ^3^ Key Laboratory of Quantum Materials and Devices of Ministry of Education School of Physics Southeast University Nanjing 211189 China; ^4^ Institute for Superconducting & Electronic Materials Australian Institute of Innovative Materials University of Wollongong Innovation Campus, Squires Way North Wollongong New South Wales 2500 Australia

**Keywords:** altermagnetism, magnetoelectric coupling, multiferroics, sliding ferroelectricity

## Abstract

Altermagnets, a recently identified class of collinear magnets, exhibit unique properties such as zero net magnetization and spin polarization dictated by lattice symmetry, making them a subject of intense research. In contrast to conventional strategies for inducing altermagnetism in antiferromagnets that rely on manipulating real‐space symmetry, this work introduces a novel and general approach to achieving altermagnetism by modulating spin‐space symmetry. Through a combination of tight‐binding models and first‐principles calculations, the microscopic origin of altermagnetism driven by spin‐space symmetry is uncovered, and the mechanism underlying enhanced spin splitting is identified. Furthermore, it is demonstrated that this spin‐space modulation can synergistically interact with ferroelectricity, enabling a spin symmetry‐dependent magnetoelectric coupling mechanism that is distinct from conventional multiferroics. This unique coupling is validated by the magneto‐optical Kerr effect, providing a robust theoretical foundation for the development of next‐generation spintronic devices that harness the potential of altermagnetism.

## Graphical abstract

Antiferromagnets can be induced into altermagnets by changing the real‐space symmetry, increasing their potential for use in spintronic devices. Here, a novel and general approach is proposed: transforming antiferromagnets into altermagnets by modulating spin‐space symmetry, rather than real‐space symmetry. This mechanism can further synergistically interact with ferroelectricity to establish a spin‐symmetry‐dependent magnetoelectric coupling framework, distinct from conventional multiferroics.

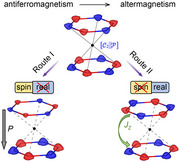

## 1 Introduction

Altermagnets, a recently discovered class of collinear magnets,^[^ ^1^, ^2^, ^3^, ^4^, ^5^, ^6^, ^7^, ^8^, ^9^ ^]^ represent a third fundamental type of magnet, distinct from conventional ferromagnets and antiferromagnets. By combining the spin polarization of ferromagnets with the fully compensated anti‐parallel magnetization characteristic of antiferromagnets, altermagnets offer unique advantages. These include enabling spintronic device responses while eliminating stray magnetic fields, which significantly enhances device stability and performance at THz frequencies.^[^ ^10^ ^]^ Consequently, altermagnets hold great potential for information storage and processing, particularly in devices leveraging the spin Hall effect or the magneto‐optical Kerr effect.^[^ ^11^, ^12^, ^13^, ^14^, ^15^ ^]^

Unlike the Rashba/Dresselhaus‐type spin splitting induced by relativistic spin‐orbit coupling in conventional antiferromagnets,^[^ ^16^, ^17^ ^]^ the spin splitting in altermagnets is governed by strict symmetry constraints described by the spin‐space group [$R_{s}∥R_{l}$],^[^ ^1^ ^]^ where transformation act on spin‐space ($R_{s}$) and real‐space ($R_{l}$). Specifically, the opposing spin sublattices in altermagnets are related not by translation (τ) or inversion (P) symmetries but by rotation (C) and mirror (M) symmetries.^[^ ^1^, ^18^ ^]^ This stringent symmetry requirements complicate the exploration of altermagnetism, especially in 2D systems. A promising strategy is to induce altermagnetism from conventional antiferromagnets through external stimuli, broadening its practical applications.

**Figure 1:**
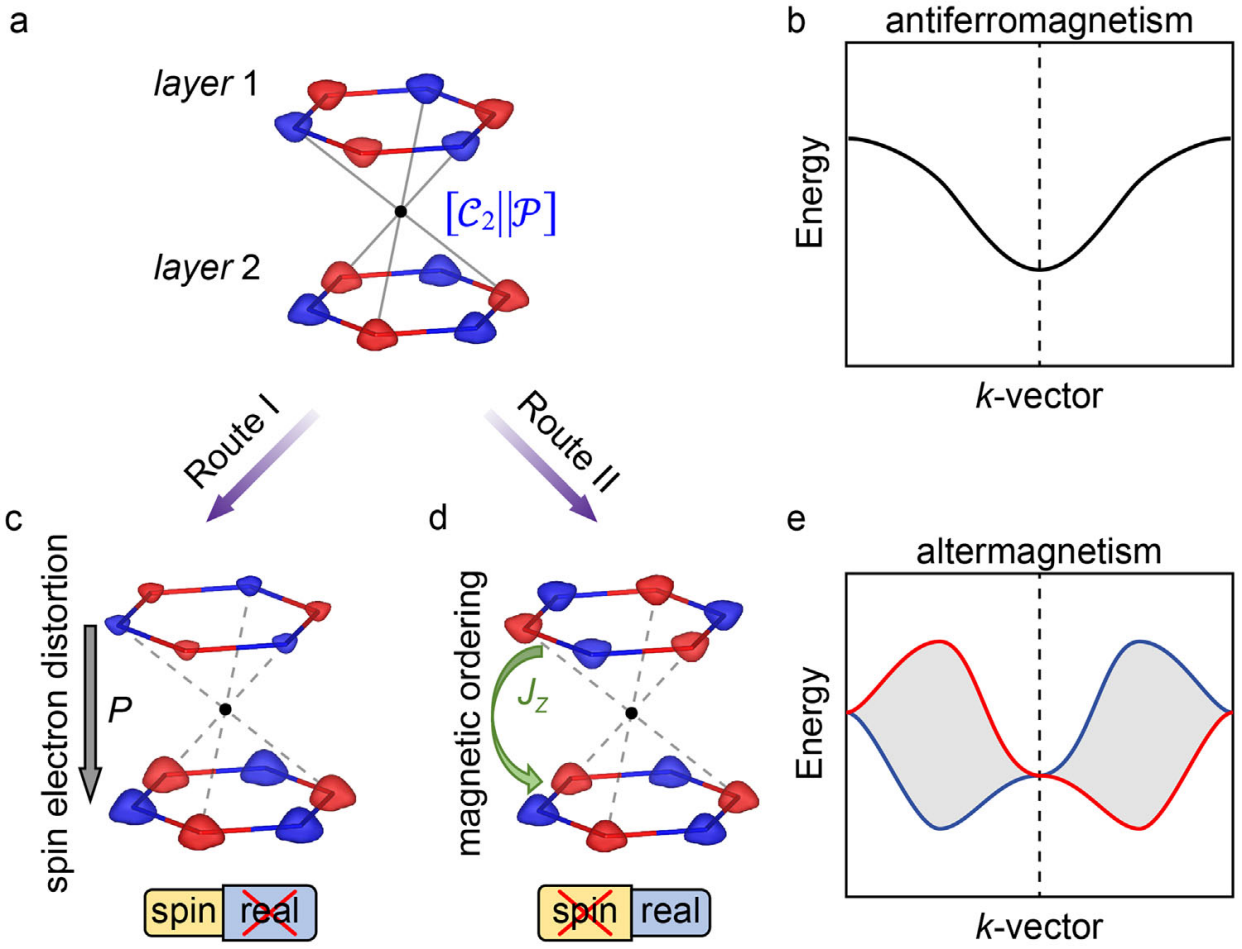
a) Schematic of an antiferromagnetic bilayer model preserving [$C_{2}∥P$] symmetry (combined spin‐ and real‐space symmetry) and its corresponding b) spin degenerate energy band structure. c) Conventional Route I for generating altermagnetism by breaking real‐space symmetry, and d) Proposed Route II demonstrating a novel mechanism for altermagnetism via breaking spin‐space symmetry. e) Characteristic spin‐split band structure of altermagnets, exhibiting momentum‐dependent spin polarization without net magnetization, contrasting with the degenerate bands in (b).

The spin degenerate bands in antiferromagnets are constrained by [$C_{2}∥\tau$] or [$C_{2}∥P$], with the band structure under the [$C_{2}∥P$] constraint illustrated in **Figure** **1**a,b. Altermagnetism can therefore be induced by breaking the symmetry responsible for spin degenerate, particularly the P symmetry in [$C_{2}∥P$], as demonstrated through external electric fields,^[^ ^18^, ^19^ ^]^ substrate‐induced effects,^[^ ^20^, ^21^, ^22^, ^23^, ^24^ ^]^ or chemical substitution.^[^ ^25^, ^26^ ^]^ This approach, which alters real‐space properties while preserving spin‐space symmetry, leads to a distortion of the spin electrons and is referred to as Route I (Figure 1c,e). Importantly, Route I can synergize with ferroelectricity, enabling magnetoelectric coupling through the mutual breaking of P symmetry.^[^ ^27^, ^28^, ^29^, ^30^ ^]^

An alternative, often overlooked, approach to breaking the [$C_{2}∥P$] symmetry involves modifying the spin‐space symmetry while preserving real‐space symmetry. This method, termed Route II (Figure 1d,e), focuses on altering magnetic ordering at spin sites such that the P symmetry no longer connects opposing sublattices. In van der Waals systems, where interlayer magnetic coupling is inherently weak and easily tunable, Route II offers a highly effective pathway. External stimuli such as strain,^[^ ^31^, ^32^, ^33^, ^34^ ^]^ doping,^[^ ^35^ ^]^ external electric fields,^[^ ^36^, ^37^, ^38^, ^39^, ^40^ ^]^ or substrate effects^[^ ^41^, ^42^, ^43^ ^]^ have been shown to modulate magnetic ordering, suggesting the strong potential for practical implementation. Notably, Routes I and II, acting in spin‐space and real space, respectively, can be combined to achieve a seamless transition from antiferromagnetism to altermagnetism.

In this work, we propose a bilayer model for inducing altermagnetism from antiferromagnetism. Through tight‐binding analysis, we elucidate the microscopic origins of spin symmetry‐driven altermagnetism. We demonstrate that spin splitting is enhanced with increasing interlayer interactions, a mechanism confirmed via first‐principles calculations in MnPTe_3_ bilayers. Furthermore, by incorporating sliding ferroelectricity, we uncover synergistic interactions between Routes I and II, paving the way for an unprecedented magnetoelectric coupling mechanism. This mechanism allows spin symmetry to govern the parity of the functional relationship between altermagnetism spin polarization direction and the ferroelectric vector. This magnetoelectric behavior, validated by the magneto‐optical Kerr effect, establishes a theoretical framework for the development of next‐generation memory devices leveraging altermagnetism. Our findings not only offer a platform for advanced spintronics applications but also broaden the scope of altermagnetic research, paving the way for future innovations in this field.

## 2 Results

### 2.1 A General Model for Altermagnetism via Tunning Spin Symmetry

**Figure 2:**
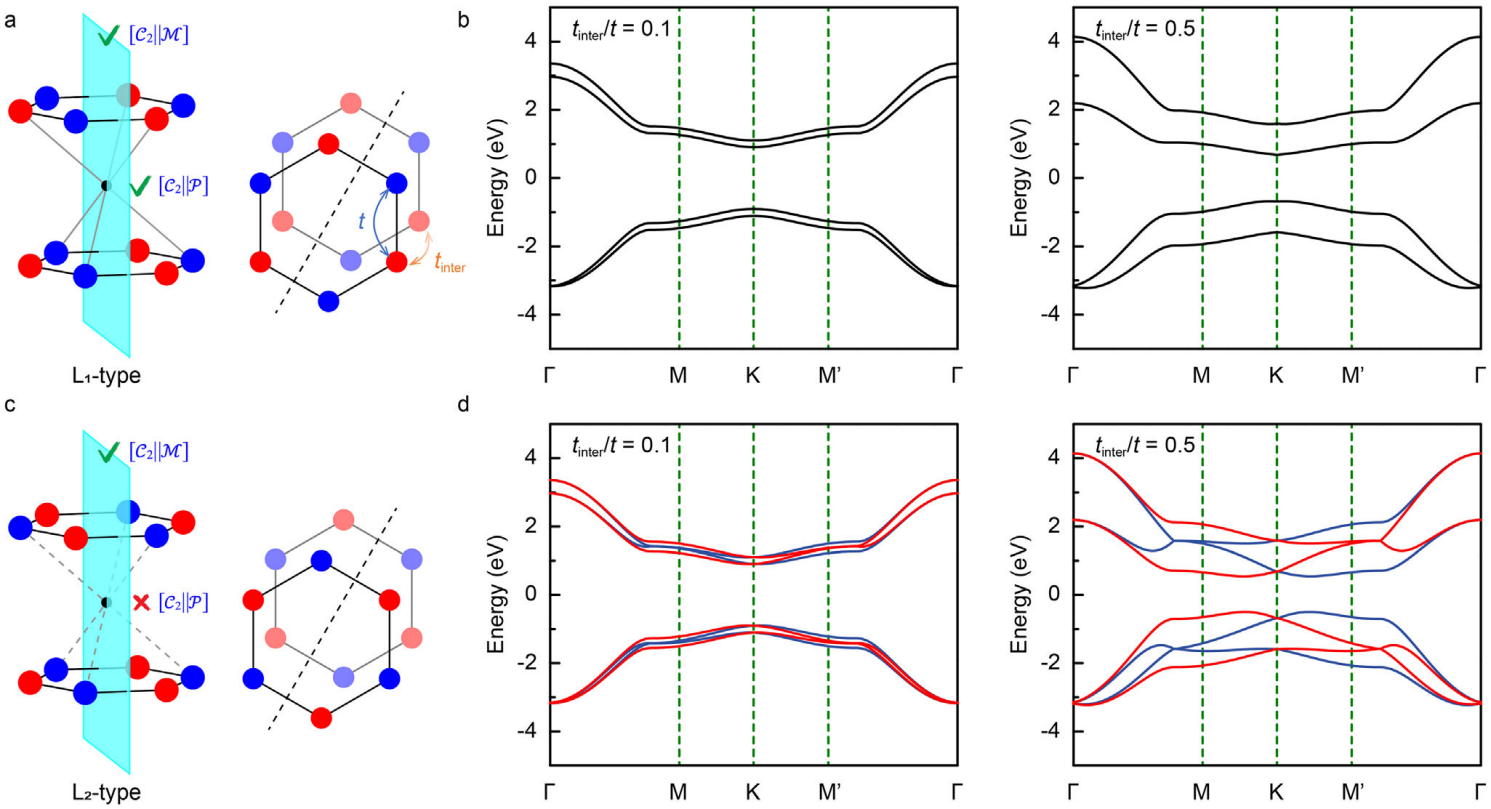
a) Bilayer lattice model with L_1_‐type magnetic ordering, and its corresponding b) spin degenerate energy band structure at different interlayer hopping strengths. c) Bilayer lattice model with L_2_‐type magnetic ordering, which has a d) spin splitting energy band structure at different interlayer hopping strengths. We used the parameters *J*_sd_ *S*/*t* = 1.

A honeycomb lattice with a Néel‐type antiferromagnetic ordering serves as an ideal model system for inducing altermagnetism due to its inherent lack of [$C_{2}∥\tau$] symmetry, enabling a focused examination of the [$C_{2}∥P$] symmetry. We analyze the bilayer system shown in **Figure** **2**, which possesses P and M symmetries in real‐space, and whose spin‐space symmetry is governed by the magnetic order. For conventional antiferromagnets with L_1_‐type magnetic ordering, both [$C_{2}∥P$] and [$C_{2}∥M$] symmetries are satisfied, leading to [$C_{2}∥P$] E(s, k) = E(‐s, ‐k) and [$C_{2}∥M$] E(s, k) = E(‐s, Mk), respectively. The latter symmetry plays a pivotal role in enabling altermagnetism. Additionally, a spin‐only symmetry $\left[{\bar{C}}_{2}||T\right]$ enforces $\left[{\bar{C}}_{2}\left|\right|T\right]E\left(s,k\right)=E\left(s,-k\right)$ for collinear magnets, where T represents time‐reversal symmetry.^[^ ^1^, ^44^ ^]^ When combined with [$C_{2}∥P$] symmetry, the [$C_{2}∥P$] $\left[{\bar{C}}_{2}||T\right]$ symmetry ensures E(s, k) = E(‐s, k), corresponding to the combined PT operation, which produces fully spin‐compensated bands. In contrast, for L_2_‐type magnetic ordering, the P symmetry connects the same spins rather than opposite ones, thereby breaking the [$C_{2}∥P$] symmetry that enforces spin degenerate. However, the remaining [$C_{2}∥M$] symmetry is expected to generate altermagnetic in the system.

To validate this hypothesis, we employ a tight‐binding Hamiltonian:

(1) $$H=t\sum_{\langle i,j\rangle ,\sigma }c_{i,\sigma }^{†}c_{j,\sigma }+t_{inter}\sum_{\langle \langle i,j\rangle \rangle ,\sigma }c_{i,\sigma }^{†}c_{j,\sigma }-J_{sd}\sum_{i,\sigma ,\sigma^{\prime }}S_{i}\cdot c_{i,\sigma }^{†}\sigma_{\sigma \sigma^{\prime }}c_{i,\sigma^{\prime }}$$

where $c_{i,\sigma }^{\left(†\right)}$ is the (creation) annihilation operator for an electron at site *i* with spin *σ*. The parameters *t* and *t*_inter_ denote the intralayer and interlayer hopping strength, respectively, while *J*_sd_ quantifies the on‐site exchange interaction between the spins of itinerant electrons and localized spins at magnetic sites *S_i_*.

Figure 2b shows the electronic spectrum for L_1_‐type magnetic ordering under Equation (1), demonstrating antiferromagnetic spin degenerate enforced by [$C_{2}∥P$]$\left[{\bar{C}}_{2}||T\right]$ symmetry. This degenerate is unaffected by interlayer hopping strength. Conversely, for L_2_‐type magnetic ordering, the broken [$C_{2}∥P$] symmetry lifts the spin degenerate, leading to spin polarization with altermagnetic characteristics, as shown in Figure 2c,d. The real‐space symmetry of the lattice remains invariant, and altermagnetism arises due to $C_{2}$ symmetry breaking in spin‐space, introduced via the layer degree of freedom. Consequently, the spin splitting increases with the stronger interlayer hopping, as depicted in Figure 2d.

### 2.2 Spin Symmetry‐Induced Altermagnetism in MnPTe_3_ Bilayer

**Figure 3:**
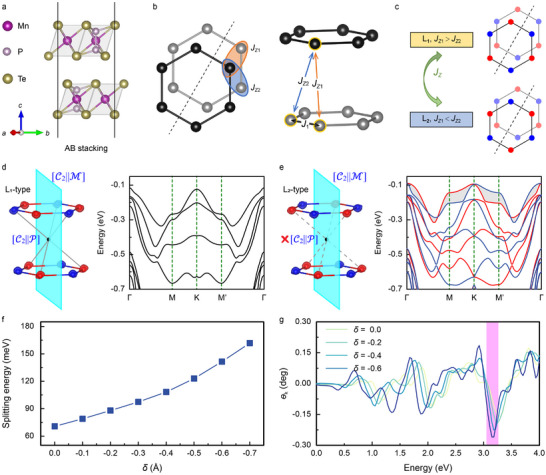
a) Side view of AB stacking MnPTe_3_ bilayer, b) highlighting the magnetic sites of Mn ions. c) Relationship between magnetic ordering and interlayer exchange interactions *J*_z_. d) and e) are the differential spin‐charge density (left) and energy band structure (right) for L_1_‐type and L_2_‐type magnetic orderings, respectively. The red (blue) sites represent spin up (spin down). To highlight the spin symmetry, the P and Te atoms in the bilayer MnPTe_3_ are hidden. f) Spin splitting energy as a function of displacement *δ*. g) Magneto‐optical Kerr effect in AB stacking.

A potential candidate material for observing altermagnetism induced by magnetic ordering is the MnPTe_3_ bilayer, an easy‐plane Néel‐type antiferromagnet (Figure S1, Supporting Information). This bilayer system comprises parallel AB stacking MnPTe_3_ monolayers (**Figure** **3**a), where magnetic sites (Figure 3b) replicate the spatial structure as the model discussed. We present in Figure S2 (Supporting Information) six different high‐symmetry configurations of bilayer MnPTe_3_. The parallel AB stacking exhibits an energy difference of 39.6 meV f.u.^−1^ compared to the ground‐state AC stacking, but the presence of a transition barrier of 24.4 meV f.u^−1^, comparable in magnitude to the sliding ferroelectric transition barrier, suggests the experimental feasibility of constructing the AB stacking as a substable state (Figure S3, Supporting Information). Intralayer atomic bonding provides robust antiferromagnetic exchange interactions *J*_1_, while interlayer van der Waals bonding facilitates magnetic ordering manipulation via external stimuli. Considering the symmetry reduction induced by stacking, the interlayer nearest‐neighbor coupling *J*_z_ degenerates into *J*_z1_ and *J*_z2_, accompanied by a minor variation of 0.02 Å in their atomic bond lengths. In this case, the magnetic ordering in the system depends on the competition between the interlayer exchange interactions *J*_z1_ and *J*_z2_. When *J*_z1_ > *J*_z2_, L_1_‐type magnetic ordering occurs; otherwise, L_2_‐type magnetic ordering arises (Figure 3c), aligning with the magnetic configurations analyzed by the tight‐binding model in Figure 2. First‐principles calculations show that the L_1_‐type magnetic ordering is stable, but the energy difference between the two configurations is only 0.95 meV, allowing for easy switching through external stimuli such as biaxial strain (Figure S4, Supporting Information). Similarly, the VPS_3_ bilayer, with an L_2_‐type magnetic ground state, provides an alternative platform, as detailed in Part 4 of Supporting Information.

Although the stacking of bilayer MnPTe_3_ inevitably introduces additional spin redistribution, the differential spin density distribution (defined as $\Delta \rho_{spin}\;\left(r\right)=\rho_{spin}^{Total}\;\left(r\right)-\rho_{spin}^{layer1}\left(r\right)-\rho_{spin}^{layer2}\left(r\right)$, where ρ_*spin*_(***r***) represents the spin density at local position *r*) revealing that the interfacial symmetric redistribution of spin‐charge preserves the P symmetry in real space, as illustrated in Figure 3d,e. In this scenario, altermagnetism can only originate from spin‐space symmetry. To further identify the spin‐splitting characteristics, the band structure displays only a subset of bands near the valence band maximum. The complete band structure encompassing conduction bands is presented in Figure S6 (Supporting Information). For the L_1_‐type magnetic ordering, the [$C_{2}∥P$] symmetry ensures complete spin degenerate. However, when the L_2_‐type magnetic ordering is induced by modulating interlayer exchange interactions, the system exhibits altermagnetic behavior, confirming that altermagnetism can be achieved by manipulating spin‐space rather than real space. To quantify the relationship between spin splitting strength and interlayer coupling, we define a displacement *δ* = *d* – *d*_0_, where *d*_0_ is the equilibrium interlayer distance. As *δ* decreases (i.e., stronger interlayer coupling), the spin‐splitting energy at the valence band maximum (Figure 3f) increases significantly.

We also calculated the magneto‐optical Kerr effect for MnPTe_3_ bilayers. This effect, a sensitive probe of T symmetry breaking and spin splitting,^[^ ^45^, ^46^ ^]^ can distinguish antiferromagnetic and altermagnetic phases. For the MnPTe_3_ bilayer with easy‐plane anisotropy, the dielectric tensor dictated by the magnetic space group *Cm* is expressed as:

(2) $$\sigma =\left[\begin{array}{ccc} \sigma_{xx} & 0 & \sigma_{xz}\\ 0 & \sigma_{yy} & 0\\ \sigma_{zx} & 0 & \sigma_{zz} \end{array}\right]$$

The Kerr rotation angle *θ*_K_(ω) is then calculated using:^[^ ^47^, ^48^, ^49^ ^]^

(3) θKω=Re−σxz−σzx2σxx1+i4πωσxx

For L_1_‐type magnetic ordering, the system exhibits no Kerr signal (Figure S7, Supporting Information). In contrast, altermagnetism with L_2_‐type ordering activates a Kerr signal, with a peak near 3.2 eV increasing alongside enhanced spin splitting (Figure 3g). These results not only demonstrate the feasibility of inducing altermagnetism through magnetic ordering but also propose a method for identifying magnetic phases using the magneto‐optical Kerr effect.

### 2.3 Spin Symmetry‐Dependent Magnetoelectric Coupling

**Figure 4:**
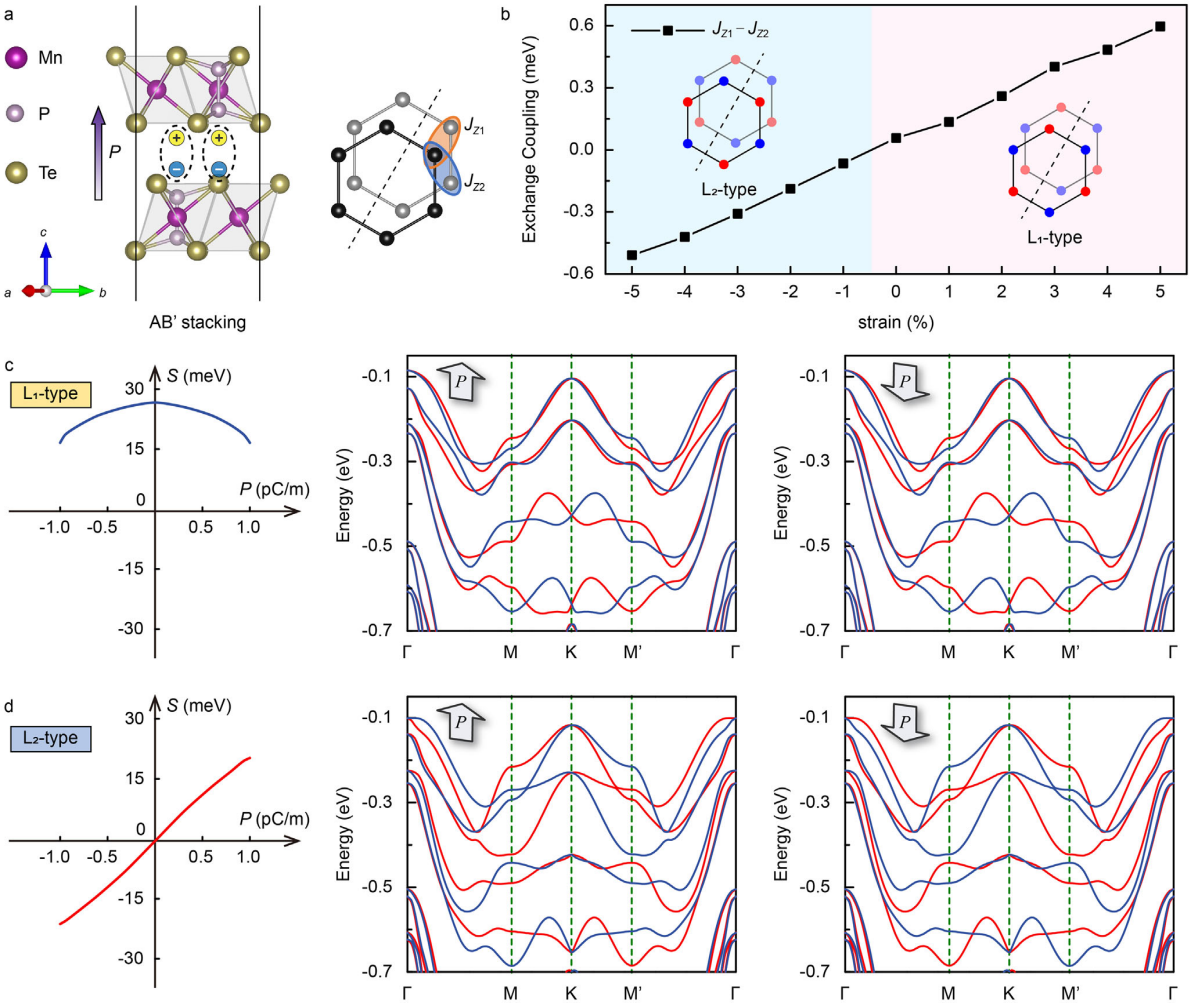
a) Side view of AB’ stacking and its corresponding magnetic sites. b) Energy difference *J*_z1_‐*J*_z2_ of interlayer exchange coupling as a function of biaxial strain. c) Altermagnetism spin splitting *S* evolution with ferroelectric P for L_1_‐type magnetic ordering, where the polarization direction switching does not change the band structure of altermagnetism. d) Altermagnetism spin splitting *S* evolution with ferroelectric P for the L_2_‐type magnetic ordering, where the altermagnetism spin splitting is inverted with polarization direction switching.

Routes I and II operate in real space and spin space, respectively, to induce altermagnetism from antiferromagnets. This mechanism can coexist within a single system and synergistically contributes to the induction of altermagnetism. Here, we employ anti‐parallel AB’ stacking in the MnPTe_3_ bilayer to generate sliding ferroelectricity. AB’ is the ground state configuration in the anti‐parallel stacking (Figure S2, Supporting Information), which breaks P symmetry without altering the spin sites, as illustrated in **Figure** **4**a. Additional details regarding the sliding ferroelectricity are provided in Figure S8 (Supporting Information). The system thus hosts two tunable order parameters: the magnetic order (L) and the ferroelectric vector (P), which act on spin‐space and real space, respectively, resulting in four distinct states. The ferroelectric vector can be reversed by the relative lateral sliding of the layers, driven by an external electric field, while the magnetic order parameter is sensitive to biaxial strain and undergoes a transition at ‐1% strain, as shown in Figure 4b. Note that since the biaxial strain does not break the [$C_{2}∥M$] symmetry, the system remains altermagnetism rather than transitioning to a fully compensated ferrimagnetism.^[^ ^50^, ^51^ ^]^

To describe the influence of ferroelectricity on spin splitting of altermagnetism, we define the altermagnetism order parameter *S*, where the positive direction of *S* is set to the direction of band splitting in the +P state, with its magnitude can be quantitatively expressed as:^[^ ^52^ ^]^

(4) $$\left|S\right|=\sqrt{\frac{1}{N_{occ}N_{k}}\sum_{k,n}{\left(\varepsilon_{n,k,↑}-\varepsilon_{n,k,↓}\right)}^{2}}$$

where *N*_occ_ is the number of occupied bands and *N*_k_ is the number of sampled *k* points. We calculated *S* as a function of P on the polarization switching path for both L_1_‐type and L_2_‐type magnetic ordering, as shown in Figure 4c,d. Note that the switching of P achieved by relative sliding between layers means that the magnitude of *S* is not only affected by P but also by the specific stacking configuration and symmetry. Therefore, we focus only on the parity of the *S*‐P function associated with the sign. For the L_1_‐type magnetic ordering, the *S‐*P relationship follows a parabolic trend, indicating that *S* is influenced by even terms of P. This is reflected in a coherent energy band structure, as shown in Figure 4c. In contrast, for the L_2_‐type magnetic ordering, the *S*‐P function is approximately linear, linking *S* to the odd terms of the P. In this scenario, switching the ferroelectric vector leads to a complete inversion of spin splitting, as shown in Figure 4d. This polarization‐induced *S* switching is equivalent to a 180° magnetic spin reversal, revealing a robust magnetoelectric coupling effect, as illustrated in Figure S9 (Supporting Information).

**Figure 5:**
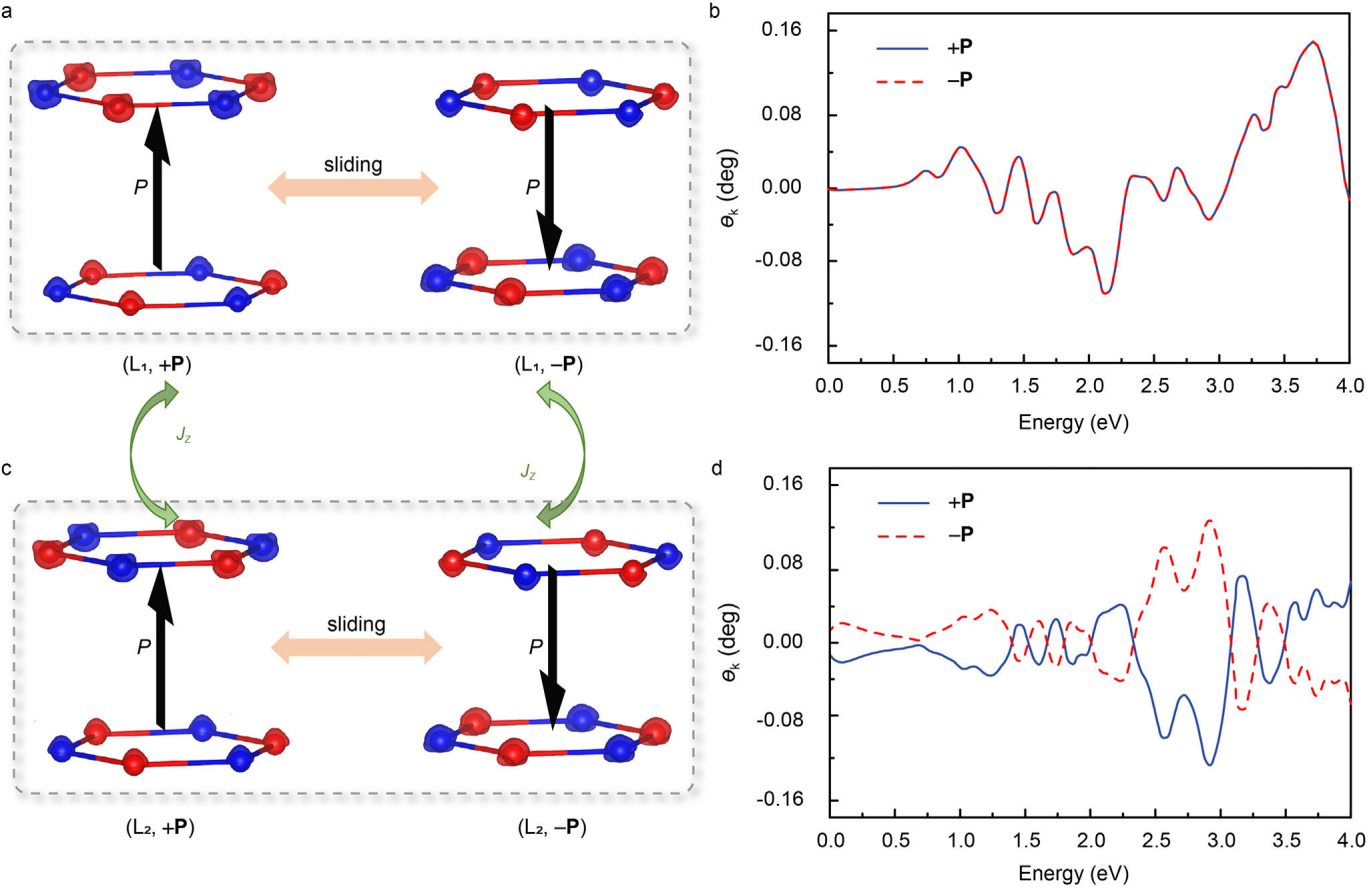
a) Effect of sliding ferroelectricity on differential spin density for L_1_‐type magnetic ordering, where b) consistent magneto‐optical Kerr signals in different polarization states show the irrelevance of altermagnetism for the polarization direction. c) represents the corresponding effect of sliding ferroelectricity for L_2_‐type magnetic ordering, where d) opposite magneto‐optical Kerr signals in different polarization states show altermagnetism depending on the polarization direction. The red (blue) sites represent spin up (spin down).

This magnetoelectric coupling, determined by spin symmetry, can be manifested by the magneto‐optical Kerr effect. **Figure** **5**a illustrates the differential spin density distribution for the L_1_‐type magnetic ordering, the asymmetric spin redistribution reveals a real‐space spin distortion induced by ferroelectric polarization, which can be further switched via relative sliding between layers. For the even function *S*‐P in L_1_‐magnetic ordering, switching the direction of P alone (while keeping its magnitude unchanged) does not affect *S*, resulting in consistent magneto‐optical Kerr signals in the +P and ‐P states, as shown in Figure 5c. However, for L_2_‐type magnetic ordering, *S* is influenced by odd terms of P, and the spin splitting reverses with the switching of polarization, leading to opposite magneto‐optical Kerr signals in different polarization states, as shown in Figure 5b,d. Additionally, the parity of the functional relationship between P and *S* in the MnPTe_3_ bilayer can be switched by altering the magnetic ordering, further highlighting the unique magnetoelectric behavior of the system.

**Figure 6:**
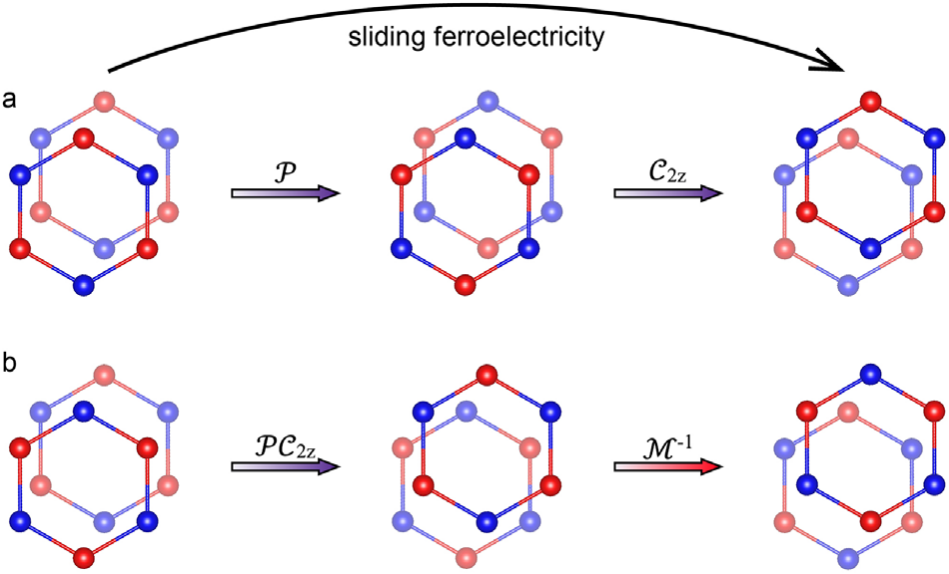
Symmetry operations correspond to sliding ferroelectric switching in the MnPTe_3_ bilayer under a) L_1_‐type and b) L_2_‐type magnetic ordering. For L_1_‐type magnetic ordering, ferroelectric switching is equivalent to the combined $PC_{2z}$. However, for L_2_‐type magnetic ordering, the magnetic ordering after the $PC_{2z}$ operation is not equivalent to that produced by actual sliding. To restore the magnetic ordering, the operation must additionally include the necessary $M^{-1}$ operation.

The spin symmetry‐dependent magnetoelectric coupling effect fundamentally originates from the unique symmetry characteristics of altermagnetism. For L_1_‐type magnetic ordering, ferroelectric polarization switching via interlayer sliding displacements does not correspond to a P‐only operation. Rather, this process requires a combined P and $C_{2z}$ operation, as shown in **Figure** **6**a. As mentioned earlier, collinear magnetic exhibits a spin‐only symmetry $\left[{\bar{C}}_{2}||T\right]$ that enforces $\left[{\bar{C}}_{2}\left|\right|T\right]E\left(s,k\right)=E\left(s,-k\right)$. This symmetry constraint renders the E(s, k) invariant under both P and τ operations, which include $C_{2z}$ and $M_{z}$ operations that are equivalent to P and τ in 2D systems. Consequently, the insensitivity of ferroelectricity to the sign of *S* stems from the invariance condition $PC_{2z}E\left(s,k\right)=E\left(s,k\right)$. For the L_2_‐type magnetic ordering, the altered spin group symmetry breaks the direct connection between opposite polarization states through simple $PC_{2z}$ operations. This modified symmetry landscape necessitates an additional $M^{-1}$ operation to relate polarization states, as shown in Figure 6b, resulting in the composite symmetry operation $PC_{2z}M^{-1}E\left(s,k\right)=E(s,-M^{-1}k$). Notably, the [$C_{2}∥M$] symmetry of altermagnetism ensures E(s, k) = [$C_{2}∥M$] E(s, k) = E(‐s, Mk). This relationship establishes that ferroelectric polarization switching becomes equivalent to the T operation acting on the magnetic spin reversal via $PC_{2z}M^{-1}E\left(s,k\right)=E(s,-M^{-1}k$) = E(‐s, ‐k) = TE(s,k), which ultimately leads to the reversal of the spin splitting in the altermagnets.

The above analysis further elucidates that the magnetoelectric coupling mechanism in altermagnetic‐ferroelectric systems has magnetic ordering tunability, which fundamentally distinguishes it from conventional multiferroics. This coupling does not require spin‐orbit coupling, but rather spin‐ferroelectric locking via altermagnetic symmetry. Crucially, the altermagnetism and sliding ferroelectricity not only coexist but are symmetrically entangled, thus overcoming the limitations of weak coupling in type‐I multiferroics and the limited applicability of type‐II multiferroics. The theory redefines the design principles of multiferroics and reveals a new class of deterministic and intrinsic magnetoelectric multiferroics.

## 3 Conclusion

In summary, we present a theoretical framework for inducing altermagnetism from antiferromagnetism by modulating spin‐space symmetries. Through a tight‐binding model analysis, we demonstrate that altermagnetism can be realized by altering magnetic ordering, a mechanism further validated by first‐principles calculations in MnPTe_3_ bilayers. This transition enables the use of the magneto‐optical Kerr effect to distinguish the distinct magnetic phases associated with altermagnetism. Furthermore, this approach can operate synergistically with sliding ferroelectricity, providing an additional pathway for inducing altermagnetism. This coexistence of these two mechanisms facilitates a novel form of magnetoelectric coupling governed by spin symmetry, representing an unprecedented coupling mechanism that is fundamentally distinct from conventional multiferroics. These findings establish a solid theoretical foundation for the exploration of altermagnetism and its potential integration into next‐generation spintronics and memory device applications.

## 4 Experimental Section

The atomic properties and electronic structure of the materials were calculated using first‐principles simulations within the framework of density functional theory (DFT).^[^ ^53^, ^54^ ^]^ The projected augmented wave pseudopotentials method, as implemented in the Vienna ab initio Simulation Package (VASP),^[^ ^55^, ^56^ ^]^ was employed. The exchange‐correlation energy was calculated using the generalized gradient approximation (GGA) of the Perdew–Burke–Ernzerhof form,^[^ ^57^ ^]^ and the plane wave cutoff energy was set to 500 eV. A Hubbard *U*_eff_ = 5 eV, with the Dudarev parametrization, was applied to accurately describe the localization of Mn 3*d* orbitals.^[^ ^58^ ^]^ The semi‐empirical DFT‐D3 method was used to account for van der Waals interaction.^[^ ^59^ ^]^ For MnPTe_3_ calculations, a centered 8 × 8 × 1 Monkhorst‐Pack *k*‐point mesh was used.^[^ ^60^ ^]^ To eliminate periodic boundary effects, the vacuum space between adjacent slabs was set to exceed 15 Å along the *z* direction. Using the conjugate gradient method, the in‐plane lattice constant and atomic coordinates were fully relaxed until the energy and force converged to 10^−6^ and 10^−2^ eV Å^−1^, respectively. The Berry‐phase method was employed to evaluate polarization magnitude,^[^ ^61^ ^]^ and the ferroelectric transition switching pathway was obtained using the climbing image nudged elastic band (CI‐NEB) method.^[^ ^62^ ^]^ The dielectric function ε_*ij*_(ω) was calculated with spin‐orbit coupling, and the optical conductivity σ_*ij*_(ω) was derived using the relation $\varepsilon_{ij}\left(\omega \right)=\delta_{ij}+i\frac{4\pi }{\omega }\sigma_{ij}\left(\omega \right)$.^[^ ^46^, ^63^ ^]^

## Conflict of Interest

The authors declare no conflict of interest.

## Supporting information



Supporting Information

## Data Availability

The data that support the findings of this study are available from the corresponding author upon reasonable request.
