# Supplementary material for: Designing Spin Symmetry for Altermagnetism with Strong Magnetoelectric Coupling
Source: Adv Sci (Weinh). 2025 Jun 17;12(30):e03235. doi: 10.1002/advs.202503235 (PMC12376617; doi:10.1002/advs.202503235)
Supplement: Supplementary file 1 — Supporting Information [file ADVS-12-e03235-s001.docx]

Supporting Information

**Designing Spin Symmetry for Altermagnetism with Strong Magnetoelectric Coupling**

*Wei Sun, Wenxuan Wang*, Changhong Yang*, Shifeng Huang*, Ning Ding, Shuai Dong, Zhenxiang Cheng**

Wei Sun, Changhong Yang, Shifeng Huang

Shandong Provincial Key Laboratory of Green and Intelligent Building Materials, University of Jinan, Jinan, 250022, China

E-mail: mse_yangch@ujn.edu.cn, mse_huangsf@ujn.edu.cn

Wenxuan Wang

School of Material Science and Engineering, University of Jinan, Jinan, 250022, Shandong, China

E-mail: mse_wangwx@ujn.edu.cn

Ning Ding, Shuai Dong

Key Laboratory of Quantum Materials and Devices of Ministry of Education, School of Physics, Southeast University, Nanjing 211189, China

Zhenxiang Cheng

Institute for Superconducting & Electronic Materials, Australian Institute of Innovative Materials, University of Wollongong, Innovation Campus, Squires Way, North Wollongong, NSW 2500, Australia

E-mail: cheng@uow.edu.au

**Part 1. The band structure and** **magnetic anisotropy energy of MnPTe_3_ monolayer.**

**
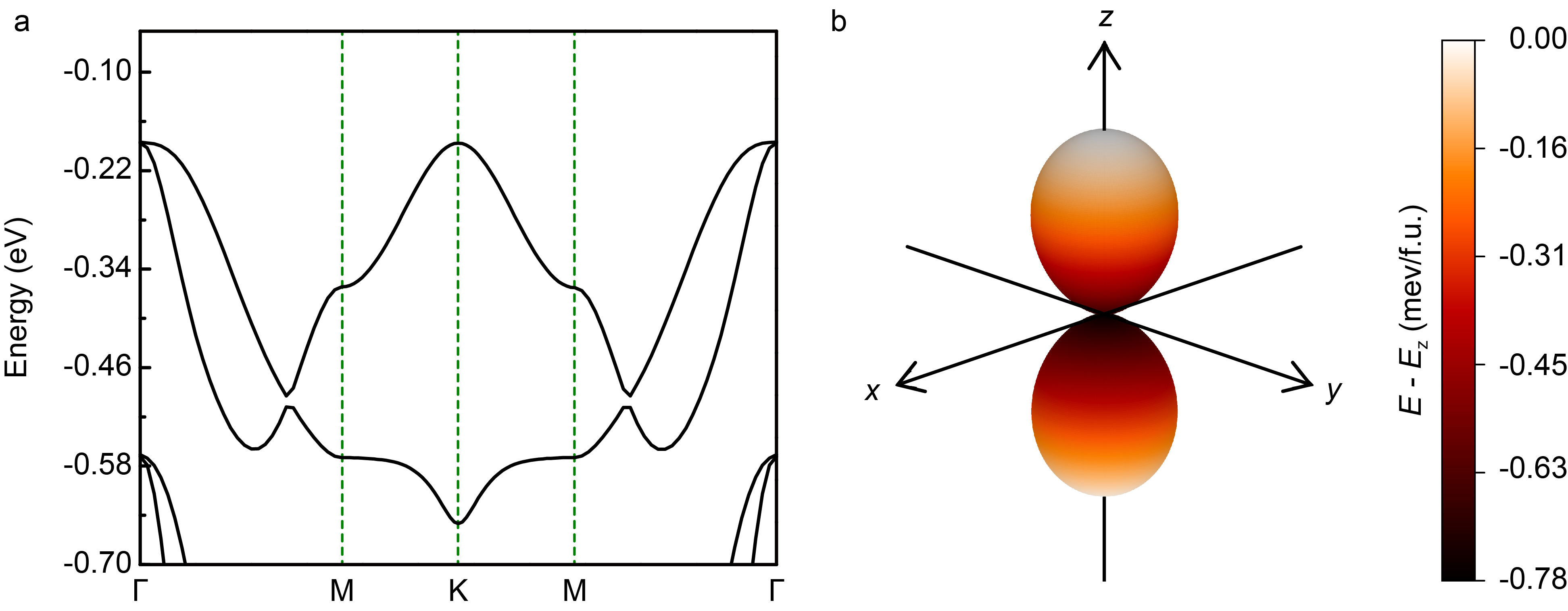
**

**Figure S1**. (a) the spin degenerate band structure of the MnPTe_3_ monolayer. (b) Angular dependence of the magnetic anisotropy energy of MnPTe_3_ monolayer with the direction of magnetization lying on the whole space, exhibiting an easy-plan magnetization.

**Part 2. Six high symmetry interlayer stacking configurations.**


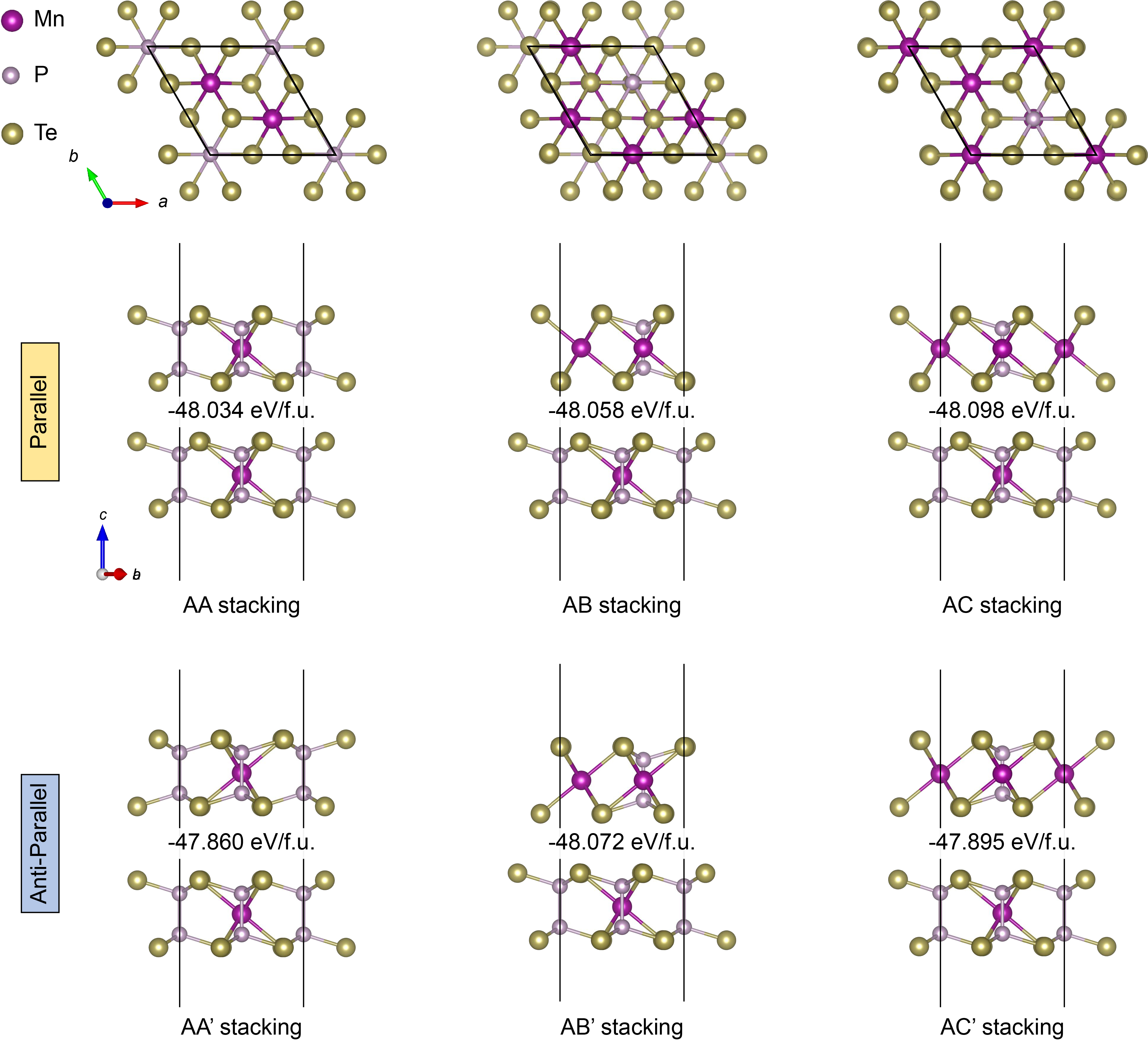


**Figure S2**. Six high symmetry interlayer stacking configurations and system energies in bilayer MnPTe_3_. The stacking configurations are categorized into two groups based on parallel and anti-parallel twist orientations. Where, AC stacking and AB’ stacking represent the ground-state stacking configurations for the parallel and anti-parallel arrangements, respectively.


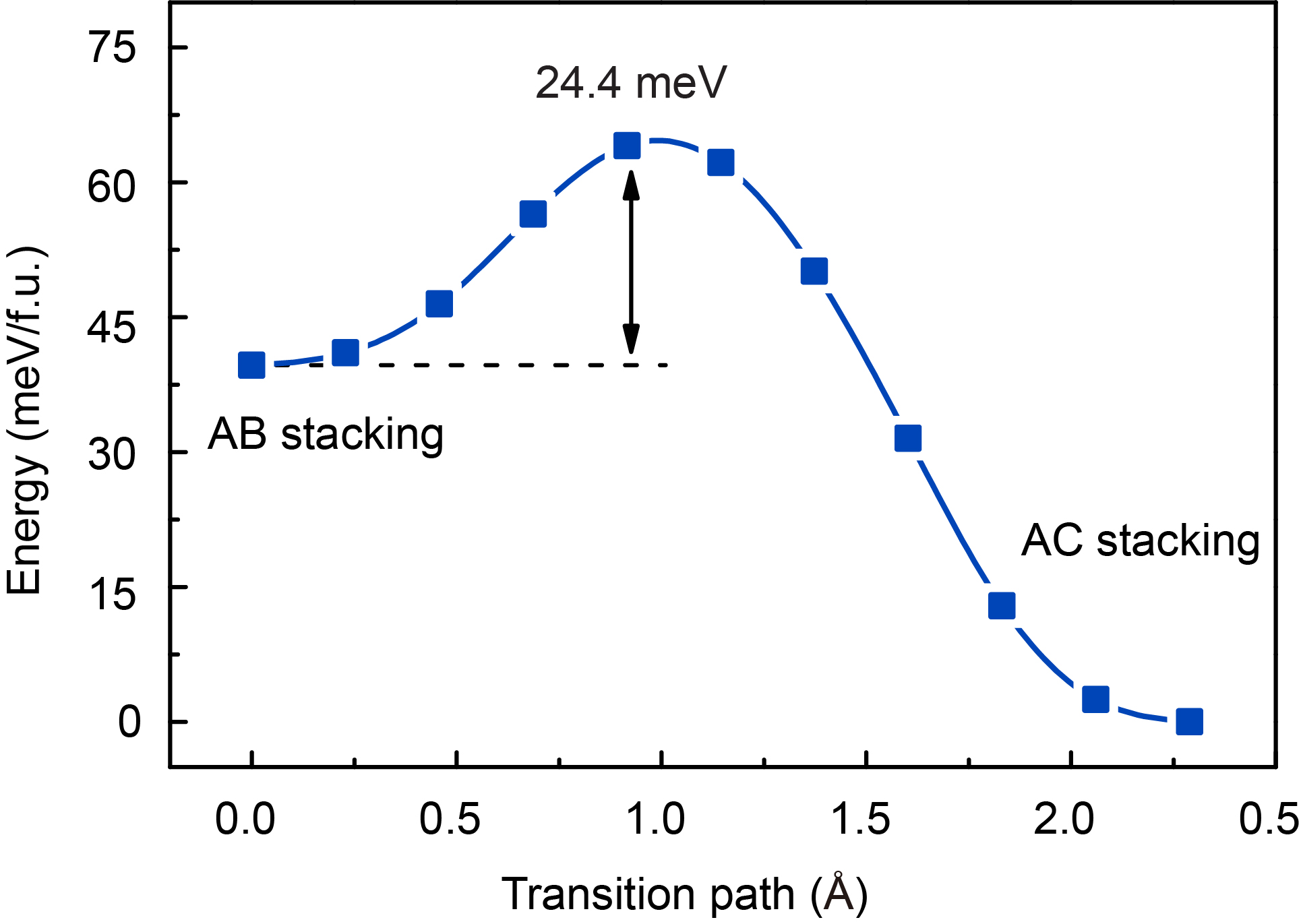


**Figure S3**. Transition barriers from AB stacking to AC stacking calculated by the climbing image nudged elastic band.

**Part 3. Spin symmetry of strain-tuned MnPTe_3_ bilayer.**


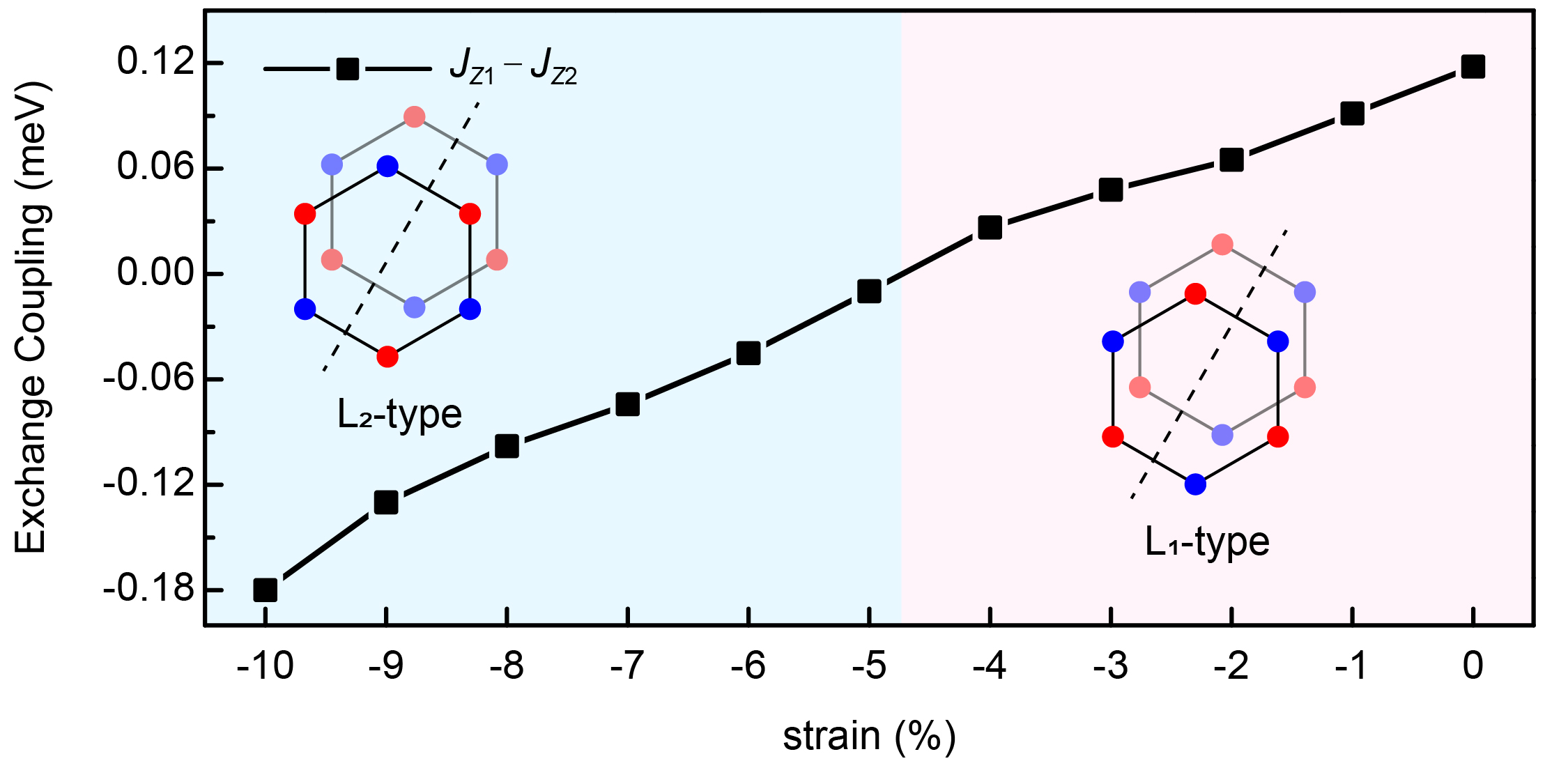


**Figure** **S4**. Energy difference *J*_z1_-*J*_z2_ of interlayer exchange coupling as a function of biaxial strain in AB stacking, which achieves a transition from L_1_-type to L_2_-type magnetic ordering at -5% strain.

**Part 4. Altermagnetism** **of VPS_3_ bilayer.**

The intralayer magnetic ordering of VPS_3_ is the same as that of MnPTe_3_, characterized by Néel-type antiferromagnetic ordering. **Figure S5**a shows the AB stacking configuration of the VPS_3_ bilayer, where the L_2_-type magnetic ordering, as determined by first-principles calculations, is 0.06 meV lower in energy than the L_1_-type magnetic ordering. Consequently, the VPS_3_ bilayer with L_2_-type magnetic ordering exhibits intrinsic altermagnetism, as shown in **Figure** **S5**b.


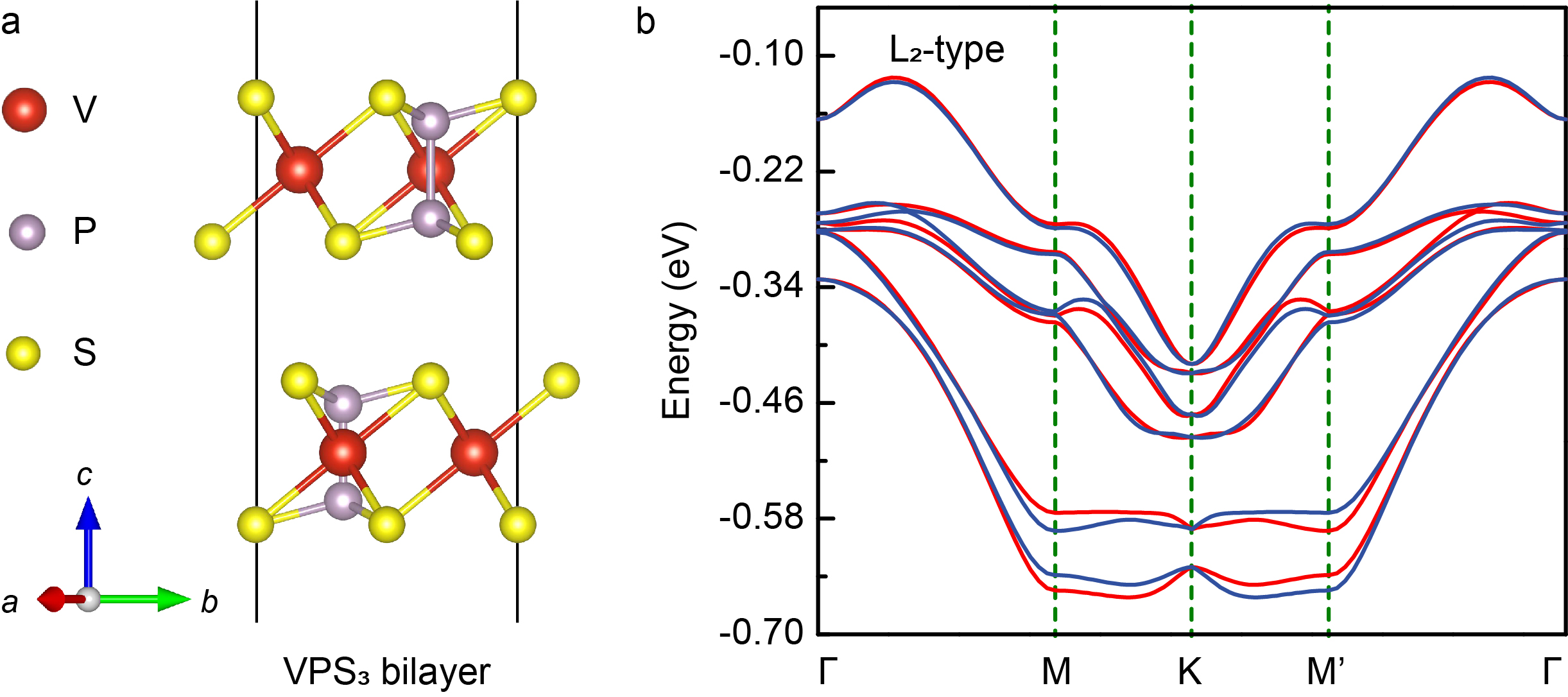


**Figure** **S5**. (a) Side view of AB stacking VPS_3_ bilayer. (b) altermagnetic band structure of the VPS_3_ bilayer with L_2_-type magnetic ordering.

**Part 5. Energy band structure for different stacking configurations and magnetic orderings.**


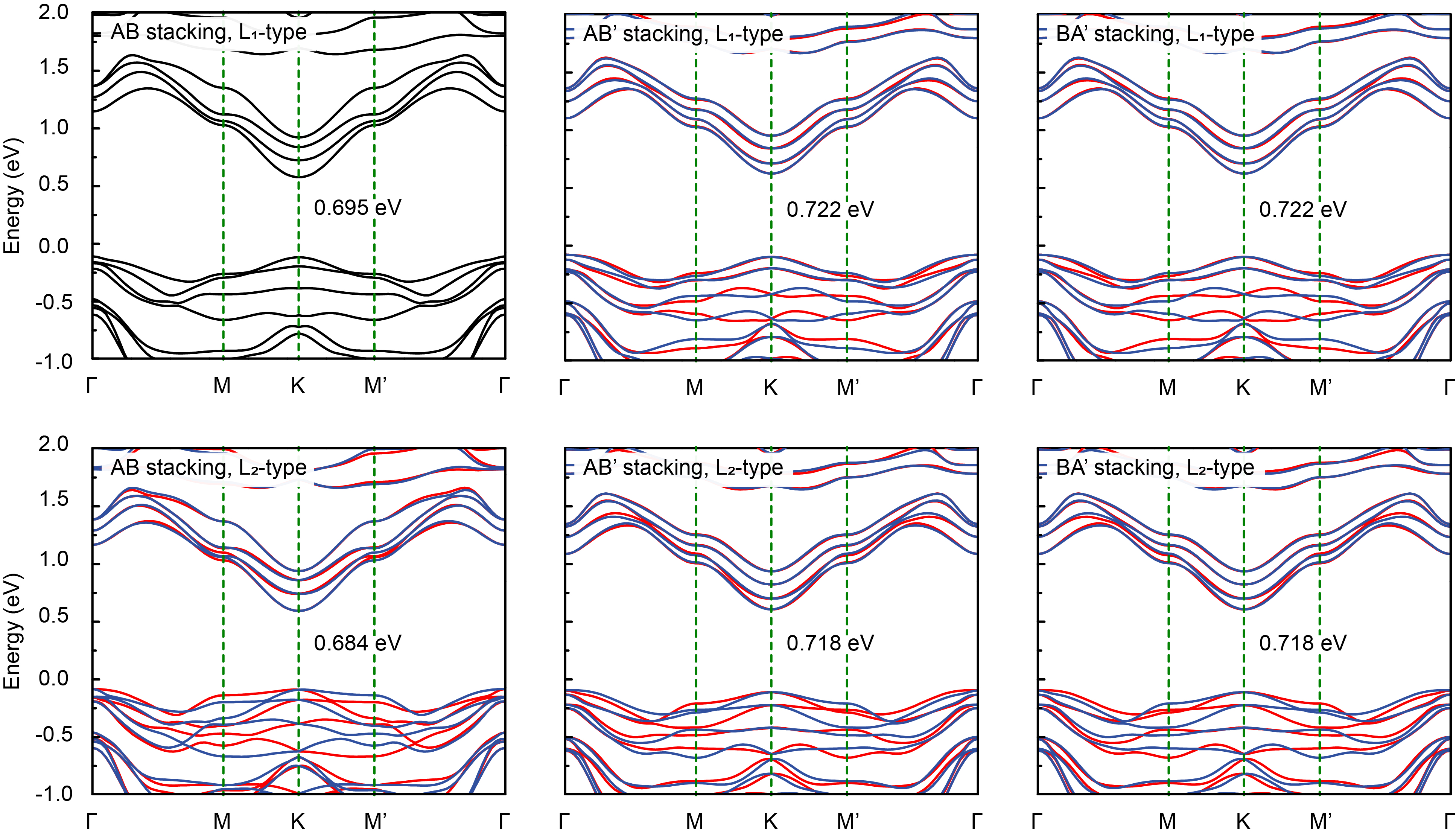


**Figure S6**. Energy band structure in the range of -1 to 2 eV for different stacking configurations and magnetic ordering, indicating that the bandgap remains robust in all configurations (within the range of 0.684 to 0.722 eV). Where, the AB’ and BA’ stacking corresponds to two different polarization states of the sliding ferroelectricity.

**Part 6. Magneto-optical Kerr effect at different spin symmetries.**


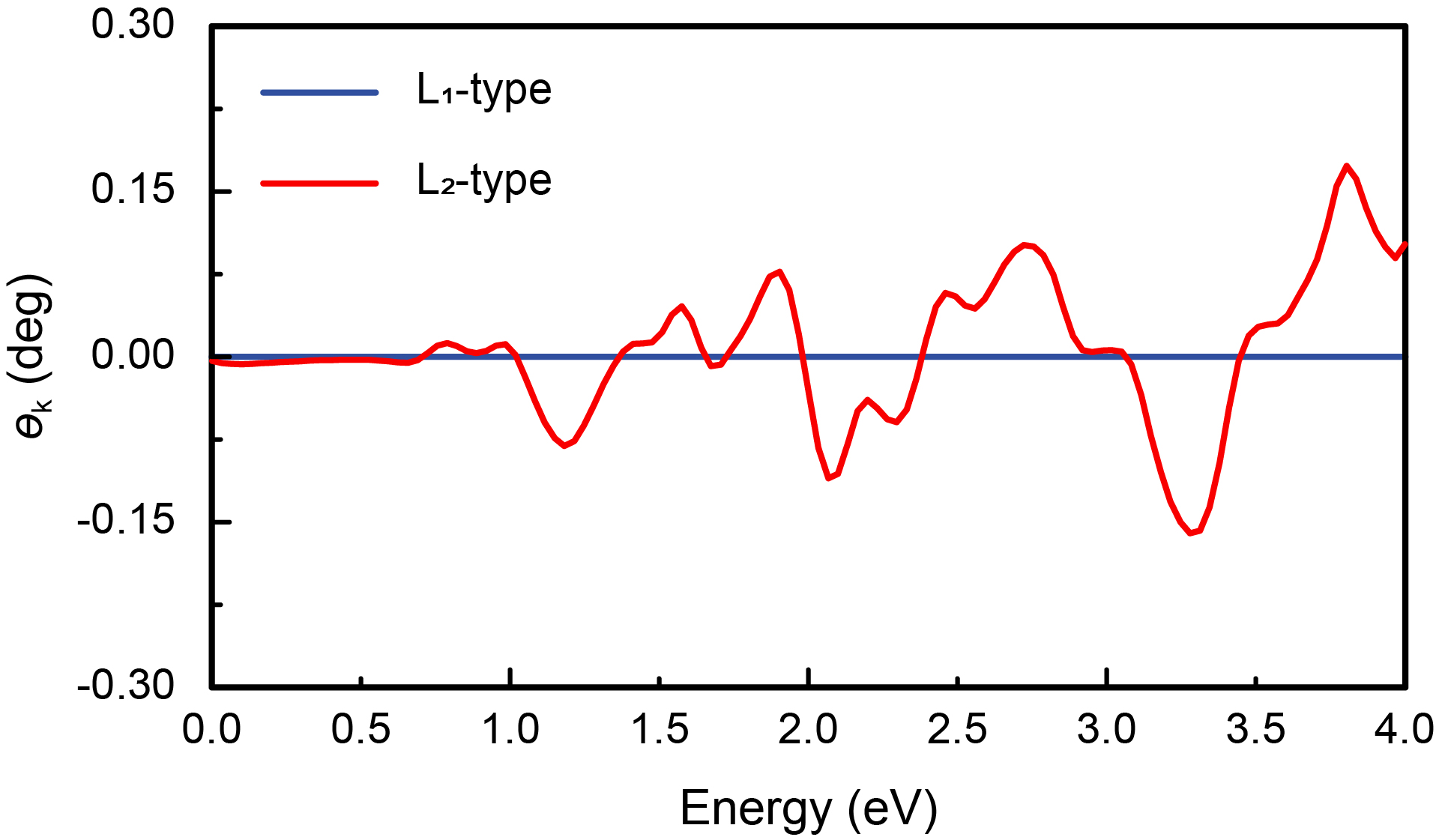


**Figure** **S7**. Magneto-optical Kerr effect at different spin symmetries. The blue lines represent conventional antiferromagnetism with L_1_-type magnetic ordering, while the red lines represent altermagnetism with L_2_-type magnetic ordering.

**Part 7. Ferroelectric properties of AB’ stacking MnPTe_3_ bilayer.**


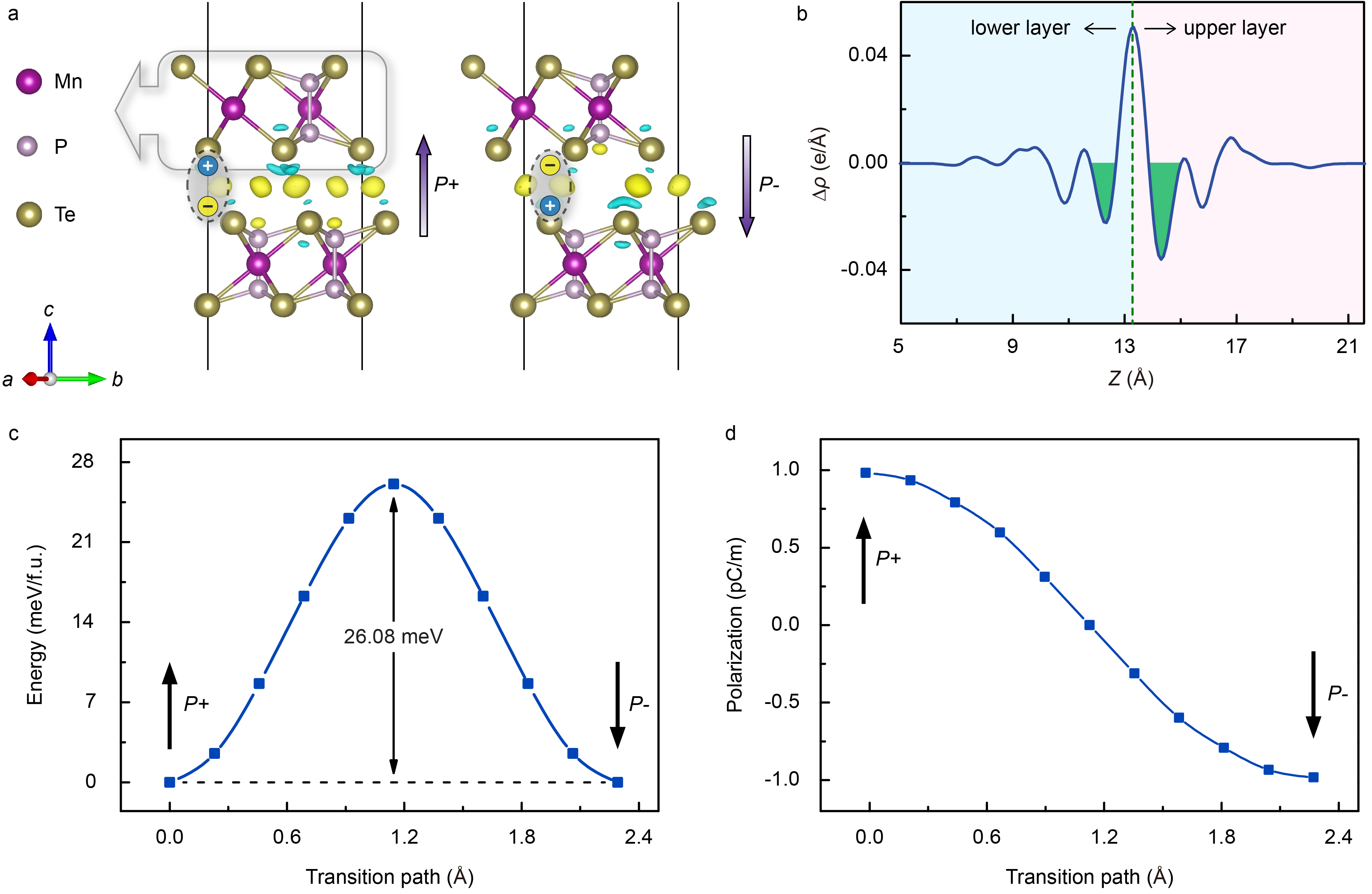


**Figure** **S8**. (a) Differential charge density of MnPTe_3_ bilayer, where the yellow and cyan regions correspond to charge accumulation and depletion. The grey box marks the direction of movement towards the opposite polarization state transition. (b) Integration of the differential charge density along the *xy* plane in *P*+ states. (c) and (d) are the transition energy barriers and polarization magnitude in the ferroelectric switching path, respectively. The arrow represents the polarization direction.

The polarization in bilayer MnPTe_3_ arises from the broken 𝒫 symmetry caused by the layer stacking. This occurs because the Te atoms at the interface between the two layers experience different environments, resulting in uncompensated interlayer vertical charge transfer and the formation of switchable out-of-plane dipole, as depicted in **Figure S8**a. The differential charge density integration along the *xy* plane, shown in **Figure S8**b, further highlights the asymmetric electron transfer at the interface, yielding an out-of-plane polarization of 0.14 pC/m. Moreover, polarization switching between the *P*+ and *P*- states can be achieved by relative lateral sliding between the layers, overcoming an energy barrier of 26.08 meV/f.u.. This demonstrates the potential for high-speed switching under a low electric field, as shown in **Figure** **S8**c-d.

**Part 8. L_2_-type magnetic ordering with magnetoelectric coupling effects in MnPTe_3_ bilayer.**


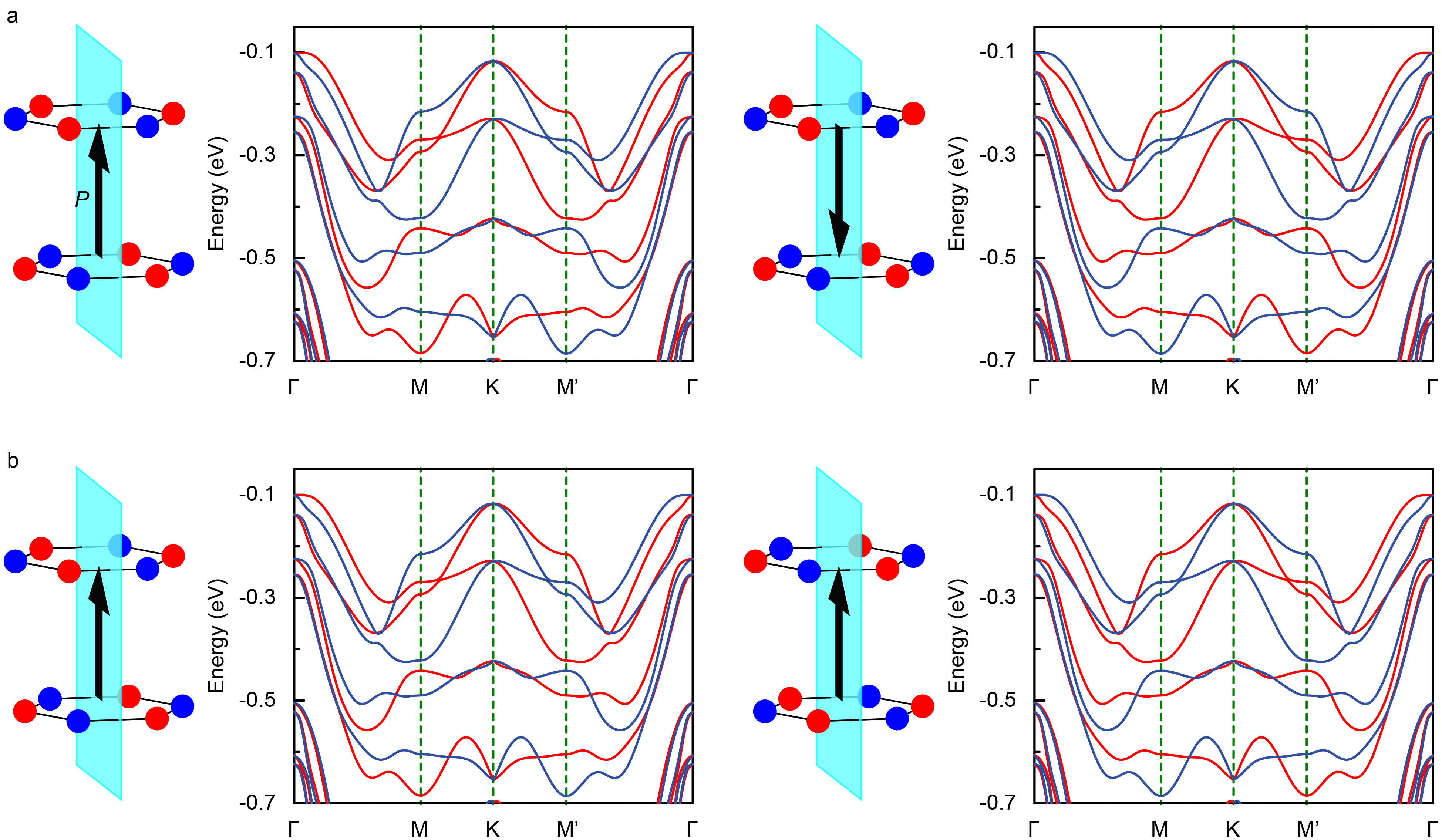


**Figure** **S9**. (a) Effect of reversed ferroelectric polarization on the band structure. (b) Effect of reversed magnetic spin on the band structure, which has the same result as reverse ferroelectricity in (a).
